# Effects of Fetal Exposure to Asian Sand Dust on Development and Reproduction in Male Offspring

**DOI:** 10.3390/ijerph13111173

**Published:** 2016-11-23

**Authors:** Seiichi Yoshida, Takamichi Ichinose, Keiichi Arashidani, Miao He, Hirohisa Takano, Takayuki Shibamoto

**Affiliations:** 1 Department of Health and Sciences, Oita University of Nursing and Health Sciences, Oita 870-1201, Japan; ichinose@oita-nhs.ac.jp; 2 Department of Immunology and Parasitology, School of Medicine, University of Occupational and Environmental Health, Fukuoka 807-8555, Japan; arashi@snow.ocn.ne.jp; 3 Environment and Non-Communicable Disease Research Center, School of Public Health, China Medical University, Shenyang 110122, China; hemiao.cmu@gmail.com; 4 Environmental Health Division, Department of Environmental Engineering, Graduate School of Engineering, Kyoto University, Kyoto 615-8540, Japan; htakano@health.env.kyoto-u.ac.jp; 5 Department of Environmental Toxicology, University of California, Davis, CA 95616, USA; tshibamoto@ucdavis.edu

**Keywords:** Asian sand dust, in utero exposure, secondary sex ratio, male reproduction, daily sperm production

## Abstract

In recent experimental studies, we reported the aggravating effects of Asian sand dust (ASD) on male reproduction in mice. However, the effects of fetal ASD exposure on male reproduction have not been investigated. The present study investigated the effects of fetal ASD exposure on reproduction in male offspring. Using pregnant CD-1 mice, ASD was administered intratracheally on days 7 and 14 of gestation, and the reproduction of male offspring was determined at 5, 10, and 15 weeks after birth. The secondary sex ratio was significantly lower in the fetal ASD-exposed mice than in the controls. Histologic examination showed partial vacuolation of seminiferous tubules in immature mice. Moreover, daily sperm production (DSP) was significantly less in the fetal ASD-exposed mice than in the controls. DSP in the fetal ASD-exposed mice was approximately 10% less than the controls at both 5 and 10 weeks. However, both the histologic changes and the DSP decrease were reversed as the mice matured. These findings suggest that ASD exposure affects both the fetal development and the reproduction of male offspring. In the future, it will be necessary to clarify the onset mechanisms of ASD-induced male fetus death and male reproductive disorders.

## 1. Introduction

Asian sand dust (ASD) storms arise from the dry and semi-arid areas of Southern Mongolia and transport dust and other particles to East China, the Korean Peninsula, Taiwan, and Japan, as well as making more than one full circuit around the globe [1,2,3,4]. Recent major epidemiological concerns about ASD are its potentially hazardous effects on child and adult asthma [5,6], allergic rhinitis [7,8], and an increase of hospitalization for pneumonia [9], as well as an increase in daily mortality in Seoul, Korea [10,11] and Taiwan [12].

We recently reported the toxic effects of sand dust on murine lungs and its aggravating effects on allergen-induced eosinophilic inflammation in the murine airway [13,14]. In addition, we have shown that ASD treatment impaired male reproductive function in mice [15].

Ambient particles are composed of various materials, including metals, organic pollutants, diesel exhaust particles (DEP), and natural sand dust, such as ASD, DEP, and carbon nanoparticles. Some particles can affect male reproductive function [16,17]. We have also shown that fetal exposure to diesel exhaust and carbon nanoparticles impairs the reproductive function of male offspring [18,19]. Another report indicates that approximately 51% of all human offspring had previously been males, but that a decline in the sex ratio at birth (or secondary sex ratio, SSR) has occurred since the middle of the 20th century [20]. Exponential regression analysis has shown a negative and significant association between particulate matter and SSR in Brazil [21]. However, the effects of fetal ASD exposure on SSR and male reproductive function have not yet been fully elucidated.

In the present study, the aggravating effects of fetal ASD exposure on SSR and the reproductive function of male offspring were investigated. After ASD was administrated to pregnant mice, sex ratio at birth, spermatogenesis, sperm characteristics, serum testosterone, serum luteinizing hormone (LH), and testicular tissue were assessed at ages 5, 10, and 15 weeks.

## 2. Materials and Methods

### 2.1. Animals

Forty pregnant CD-1 mice purchased from CLEA Japan Inc. (Tokyo, Japan) were used—induction at day five post-coitum (p.c.; the day the plug was found was considered day 0 p.c.). Mice were divided into two groups of 20 mice each: the nanoparticle and control groups. Up to day 16 of induction, each cage housed five mice, and starting on day 17 of induction, each mouse was caged separately. The mice were kept under the following conditions: temperature 23–25 °C, humidity 50%–70%, and 12-h light/dark cycle. Mice had free access to food (CE-2 solid food, CLEA Japan) and water. Animals were treated in accordance with the guidelines of Oita University of Nursing and Health Sciences. The experiments were carried out under the approval of the Committee of Research Ethics and Safety Commission of the Oita University of Nursing and Health Sciences (approval number: 764, 2013).

### 2.2. ASD Particles

ASD was collected from the atmosphere at the University of Occupational and Environmental Health, Kitakyushu, Fukuoka, Japan on 1–3 May 2011, after massive dust storm events in East Asia. Collection was carried out using a high-volume air sampler (Sibata Scientific Technology, Saitama, Japan) with a Teflon filter. The density ranges of the ambient particulate matter by Light Detection and Ranging (LIDAR) on 1–3 May was 350–550 μg/m^3^ in Nagasaki (Nagasaki Prefectural Institute of Public Health and Environmental Science), Japan. The collection flow was 770 L/min, and the instrumental classification size (defined as the 50% cut-off size of the aerodynamic diameter) was 1st stage (5.9 μm), 2nd stage (2.8 μm), 3rd stage (1.7 μm), 4th stage (0.91 μm), and back-up (<0.91 μm), respectively. The top peak in the mass size distribution for ASD ranged from 0.91 to 1.7 μm. The collected ASD was stored at −30 °C in a germ-free case with a desiccant until use.

The concentration of each element (Si, Na, Mg, Al, P, Ca, Ti, Cr, Mn, Fe, Cu, Ba, and Sr) was determined by inductively-coupled plasma atomic emission spectroscopy (ICP-AES, 61E Trace and ICP-750, Thermo Jarrell-Ash Co., Midland, ON, Canada) after acid digestion with mixed acids (68% nitric, 38% hydrofluoric, and 70% perchloric = 5:1:1) was performed on a sample at 180 °C for 3 h. The chemical elements in ASD were 71.0% SiO_2_, 13.0% Al_2_O_3_, 5.4% Fe_2_O_3_, 2.5% CaO, 3.5% CaCO_3_, 3.0% MgO, and 0.6% TiO_2_.

### 2.3. Particle Administration

The concentration of the ASD solution was adjusted to 2 mg/mL using physiological saline (Otsuka Co., Kyoto, Japan). The solution was then sonicated for 5 min prior to administration. On days 7 and 14 of gestation, 200 μg was administered intratracheally. Exposure began after implantation and initial organ development had taken place.

### 2.4. Offspring

Mice delivered after a gestation of 18.0 to 19.0 days were used. At the age of 12 days, six male mice (from each mother mouse) were selected at random. Finally, 29 dams—including 16 controls and 13 ASD—were used, and male offspring were randomly selected from different dams. Mice were weaned at 25–26 days after birth and removed from their mothers’ cages. Each cage of young males housed six to eight mice. Finally, 86 male offspring, including 48 controls and 38 ASD, were used.

### 2.5. Testicle and Epididymis Removal

At age 5 (puberty), 10 (young adult), or 15 (adult) weeks, male mice were anesthetized using pentobarbital sodium. After body weight was measured, a blood sample was collected from the heart. Mice were sacrificed to remove testicular and epididymal tissues. Both left and right testicle tissues were separately weighed. The right testis was stored frozen at −80 °C and was used to measure daily sperm production (DSP). The right epididymis was used to evaluate sperm characteristics. The left testis was used for histological analysis.

### 2.6. Sperm Characteristics

The tail of the epididymis in 1 mL of 10% fetal bovine serum (FBS) (Sigma-Aldrich Japan, Tokyo, Japan) M199 medium solution was chopped one time using ophthalmological scissors and filtered using 100-mesh membrane to obtain a sperm suspension. The number of spermatozoa in the tail of the epididymis was counted using a cytometer. In addition, smear samples were prepared to observe spermatozoa. Abnormal spermatozoa were defined as cells without heads or tails, and the ratio of abnormal spermatozoa was calculated. A sperm analyzer (SCA, NeuroScience, Tokyo, Japan) was used to determine the concentration of sperm in the epididymis, and to assess sperm motility. Approximately 800–1000 spermatozoa were analyzed in each sample.

### 2.7. Testicular Daily Sperm Production (DSP) 

A lysis buffer containing 8.8 g/L sodium chloride and 200 mL/L 0.02% eosin-Y solutions were used for DSP measurement. The testis was homogenized in 1 ml of lysis buffer and was stored at 4 °C. The number of cells (spermatozoa) in the solution was counted to calculate DSP using the following formula [22]:

Sperm count/mL × volume of lysis buffer = testicular sperm count

Testicular sperm count/4.84 * = sperm produced/day

(Sperm produced/day)/testis weight = DSP

*: 4.84 is a coefficient for calculating sperm production in mice.

### 2.8. Serum Testosterone Measurement

Serum testosterone and luteinizing hormone (LH) were measured using the Testosterone ELISA Test Kit (Endocrine Technologies Inc., Newark, CA, USA) and the LH ELISA Test Kit (Endocrine Technologies Inc., Newark, CA, USA).

### 2.9. Histological Analysis

A testis prepared in Bouin’s solution was embedded in paraffin and then thin sliced. After a slice was stained using hematoxylin and eosin (HE) according to the usual method, it was observed under a Nikon ECLIPSE light microscope (Nikon Co., Tokyo, Japan). Variables assessed included seminiferous epithelium damage, vacuolation of seminiferous tubules and interstitial edema.

### 2.10. Statistical Analysis

All data are presented as means ± standard deviations (SDs). All statistical analyses were performed using KyPlot version 5 (Kyens Lab Inc., Tokyo, Japan) for statistical analysis. Chi-Square Test was used to analyze gestation length, litter size, fertility, and gender ratio. Welch’s *t*-test was used to analyze body weights, organ weights, sperm production, and hormone concentration. A *p*-value < 0.05 was considered to represent statistical significance.

## 3. Results

### 3.1. Effects of ASD Administration on Maternal and Fetal Growth

The effects of ASD administration on fertility parameters and pups were determined by comparing gestation length, litter size, fertility, and gender ratio between the two groups. The sex ratio differed significantly between the ASD and control groups (Chi-Square Test: *p* < 0.05). ASD treated females gave fewer sons (42.9%) than the control females (54.2%). However, no significant differences were observed in gestation length, litter size, or fertility (Table 1).

### 3.2. Effects of Fetal ASD Exposure on Body, Testis, and Epididymis Weights

Testicular weight was significantly decreased in the ASD treated group at 5 weeks (86.3%, *p* < 0.01) (Table 2). Epididymis weight was considerably decreased in the ASD treated mice at 5 and 15 weeks (88.8%, *p* < 0.05 at 5 weeks; 89.8%, *p* < 0.05 at 15 weeks) (Table 2). There were no significant differences in body weight between the ASD and control groups at 5, 10, or 15 weeks (Table 2).

### 3.3. Histological Changes in the Testes of Mice Exposed to ASD During the Fetal Period

**Figure 1:**
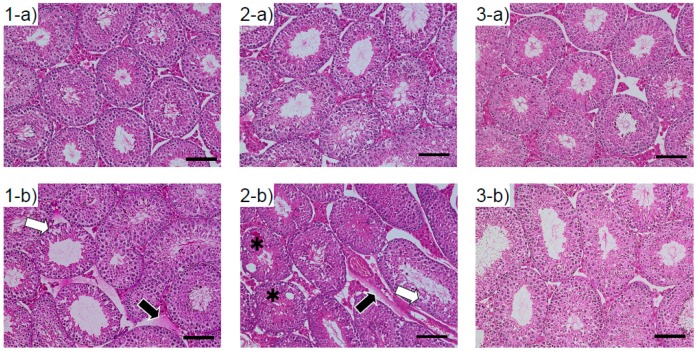
Testicular tissue of male offspring exposed to ASD as fetuses. **1**: 5-week-old mouse; **2**: 10-week-old mouse; **3**: 15-week-old mouse. **a**: control; **b**: ASD group, ×200 magnification. H&E stain. At 5- and 10-weeks-old, mice in the ASD-exposed group showed seminiferous epithelial damage (white arrows). Interstitial edema was observed in the testes of fetal ASD-exposed mice at 5 and 10 weeks (black arrows). Vacuolation was seen with some seminiferous tubules *. These observations were found in other animals (N = 6, each group).

The testes of male mice exposed to ASD as fetuses exhibited vacuolation of the seminiferous tubules (asterisks) and low cellular adhesion of seminiferous epithelia (white arrows) (Figure 1). Interstitial edema was observed (black arrows) in the ASD treated group at 5 and 10 weeks.

### 3.4. Effects of Fetal ASD Exposure on Sperm Production

**Figure 2:**
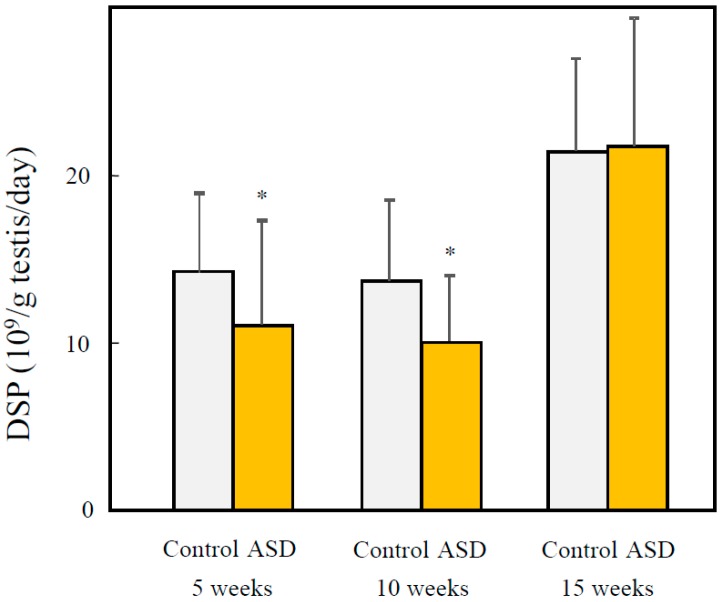
Effects of fetal ASD exposure on spermatogenesis. A right testis sample was weighed, and seminiferous tubules were released from the tunica albuginea into a saline solution in a homogenizer tube. The tissue was homogenized, diluted to the desired volume, and a sample of the diluted suspension was taken to count sperm heads or sperm cells in a haemocytometer chamber. DSP was calculated as described in Methods. N = 16, 16, 16, 12, 13, and 13 for 5, 10, and 15 weeks control, 5, 10, and 15 weeks ASD, respectively. * *p* < 0.05, vs. controls. DSP: daily sperm production.

DSP was significantly decreased in the ASD treated group at 5 and 10 weeks (72.0%, *p* < 0.05 at 5 weeks; 73.2%, *p* < 0.05 at 10 weeks) (Figure 2). On the other hand, there were no significant differences observed between the ASD and control groups at 15 weeks.

### 3.5. Effects of ASD on Serum Testosterone and LH

**Figure 3:**
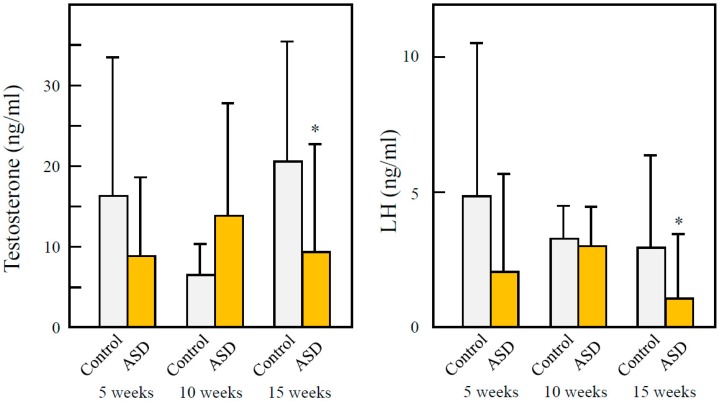
Effects of fetal ASD exposure on serum testosterone and serum luteinizing hormone (LH) concentration. Testosterone and LH levels in serum were determined by ELISA. Means ± SD. N = 16, 16, 16, 12, 13, and 13 for 5, 10, and 15 weeks control, 5, 10, and 15 weeks ASD, respectively. * *p* < 0.05, vs. controls.

ASD tends to decrease serum testosterone and LH at 15 weeks after birth. However, there were no significant differences in serum testosterone or LH between ASD and control groups at either 5 or 10 weeks (Figure 3).

## 4. Discussion

Our previous study investigated the effects of fetal ASD exposure on male reproductive functions. However, the effects on male offsprings’ reproductive function have not been studied. In the present study, the effects of ASD exposure in utero were examined—specifically changes in the sex ratio (that is, reduction in the proportion of male offspring) at birth and the reproductive functions of the male offspring produced.

Previous reports have indicated that the population of newly born human males declined significantly in several countries in the 20th century, including Canada [23], the United States, and Japan [24]. This suggests that these declines in SSR may be related to prenatal exposures to environmental pollutants (such as air pollution) at a critical stage in utero sexual differentiation and/or increased reabsorption of male embryos. In a previous study, fetal ASD exposure significantly decreased SSR. Since we administered ASD at days 7 and 14 of gestation, the loss of male offspring was evident by abortion or embryonic death of male fetuses. Thus, possible causes for the SSR decline include factors affecting abortion and embryonic death.

Fetal ASD exposure also significantly reduced the DSP in male offspring that were produced. When carbon nanoparticles were administered to pregnant mice, DSP decreased significantly in male offspring [19]. Therefore, carbon nanoparticles and ASD reduce the DSP through fetal exposure. It has also been reported that fetal exposure to diesel exhaust (DE) lowers the DSP of male offspring [25]. DE consists of various components, including large amounts of particles. Therefore, fetal DE exposure and fetal carbon nanoparticles may lower DSP in male offspring due to component particulate matters in the same way that exposure to ASD does. However, in the present study, the components of DE and carbon nanoparticles were different from ASD. In the future, it will be necessary to determine the effects of fetal exposure to the components of ASD and carbon nanoparticles on male offspring.

Intercellular adhesion of seminiferous epithelia was observed in the testes of younger male offspring. The low cellular adhesion of seminiferous epithelia may indicate a reduction of Sertoli and spermatogenic cell adhesion. As Sertoli cells supply nutrients and send signals for cellular differentiation to spermatogenic cells [26], low adhesion of Sertoli and spermatogenic cells may hinder spermatogenesis, and thus reduce DSP in younger male offspring with fetal ASD exposure. However, there were no observations of this in the testes of older male offspring. These findings accorded with the reduction of DSP. When pregnant mice were exposed to carbon nanoparticles, lowered cellular adhesion and DSP were observed in both immature and mature male offspring [19]. In the present study, however, when pregnant mice were exposed to ASD, testicular damage and reduction of DSP occurred in the immature male offspring, but the mice recovered with maturation. Serum testosterone and LH concentration operated in the opposite manner: there were no significant elevations observed in the immature males, but elevated levels were observed in the mature mice. These findings suggest that there is no relationship between a reduction of DSP and testicular damage with serum sexual hormone concentrations. Moreover, it is suggested that improving the air pollution contributes to a normalization of SSR and an improvement in male reproduction.

## 5. Conclusions

The results of the present study suggest that fetal ASD exposure reduces DSP and induces seminiferous tube damage in immature male offspring, but that they recover with sexual maturation. In addition, fetal ASD exposure significantly depressed the ratio of male to female offspring.

## Figures and Tables

**Table 1:** Effects of Asian sand dust (ASD) exposure on fertility, gestation length, litter size, and sex ratio at birth.

Variable	Control	ASD
Fertility (%)	95%	80%
Gestation length (day)	18.6 ± 0.2	18.8 ± 0.3
Litter size	14.6 ± 1.9	13.3 ± 3.6
Sex ratio at birth	0.542	0.429 *

* *p* < 0.05.

**Table 2:** Effects of fetal ASD exposure on offspring body, testis, and epididymis weights.

Weeks	Pups Number		Body Weight (g)		Testis (mg)		Epididymis (mg)	
	Control	ASD	Control	ASD	Control	ASD	Control	ASD
5	16	12	33.9 ± 2.8	33.0 ± 3.6	118.2 ± 9.3	102.1 ± 25.0 **	31.3 ± 4.5	27.8 ± 4.0 *
10	16	13	43.7 ± 3.2	44.8 ± 2.4	145.7 ± 23.8	147.9 ± 11.4	54.0 ± 5.3	54.8 ± 4.8
15	16	13	46.4 ± 3.4	46.9 ± 4.6	159.9 ± 27.7	147.2 ± 36.3	64.5 ± 7.2	57.9 ± 8.1 *

* *p* < 0.05; ** *p* < 0.01.

## Abbreviations

The following abbreviations are used in this manuscript:

ASD	Asian sand dust
DE	Diesel exhaust
DEP	Diesel exhaust particles
DSP	Daily sperm production
HE	hematoxylin and eosin
ICP-AES	inductively-coupled plasma atomic emission spectroscopy
LH	luteinizing hormone
LIDAR	Light Detection and Ranging
p.c.	post-coitum
SSR	Secondary sex ratio
